# BioID data of c-MYC interacting protein partners in cultured cells and xenograft tumors

**DOI:** 10.1016/j.dib.2014.10.001

**Published:** 2014-10-14

**Authors:** Pak-Kei Chan, Tharan Srikumar, Dharmendra Dingar, Manpreet Kalkat, Linda Z. Penn, Brian Raught

**Affiliations:** Princess Margaret Cancer Centre, University Health Network, and Department of Medical Biophysics, University of Toronto, Toronto, ON, Canada

## Abstract

BioID was performed using FlagBirA⁎ (the R118G biotin ligase mutant protein) and FlagBirA⁎-Myc in HEK293 T-REx cells maintained both under standard cell culture conditions and as mouse xenografts. The mass spectrometry dataset acquired in this study has been uploaded to the MassIVE repository with ID: MSV000078518, and consists of 28 ⁎.raw MS files acquired on an Orbitrap Velos instrument, collected in data-dependent mode. iProphet processed MS/MS search results are also included as a reference. This study has been published as “BioID identifies novel c-MYC interacting partners in cultured cells and xenograft tumors”, by Dingar et al. in the Journal of Proteomics, 2014 [1].

**Specifications Table**

**Table:** 

Subject area	Biology
More specific subject area	Proteomics, Protein–protein interactions
Type of data	Mass spectrometry RAW files
How data was acquired	Mass Spectrometry (Thermo Orbitrap Velos)
Data format	RAW unprocessed files
Experimental factors	Bait protein maintained under standard culture conditions and as xenografts
Experimental features	BioID using the human c-MYC protein as bait
Data source location	MassIVE
Data accessibility	Available on MassIVE, ID: MSV000078518

**Table:** **Value of the data**.

• The first *in vivo* BioID • The first BioID-based c-MYC interactome • >100 MYC interactors identified (>30 previously described)

## 1 Data, experimental design, materials and methods

### 1.1 In vivo BioID

6–8 week old NOD-SCID male mice were used for the study as per institutional guidelines. Two million FlagBirA⁎ or FlagBirA⁎-MYC 293 cells were injected into the left and right flanks of mice (6–8 weeks, NOD-SCID male mice). On the same day, Myc fusion protein expression was induced with tetracycline hydrochloride (Sigma) in drinking water (2 g/liter) until the experimental end point. Tumors were measured with calipers, and upon reaching ~800 mm^3^ mice were injected intraperitoneally with 30 ug biotin (0.15 mg/ml in PBS) once per day for two days. On the third day, mice were anesthetised with isofluorane, sacrificed, and xenografts removed and flash frozen. Frozen xenografts were pulverized with a mortar and pestle, and the powder resuspended in ice-cold modified RIPA buffer at 1:5 (w:v; 1% NP-40, 50 mM Tris–HCl pH 7.5, 150 mM NaCl, 1 mM EDTA, 1 mM EGTA, 0.1% SDS, 1:500 protease inhibitor cocktail (Sigma) 0.5% sodium deoxycholate), solubilized in a dounce tissue homogenizer (Kontes), and incubated with 250 U benzonase nuclease (EMD) for 1 h at 4 °C. The solution was sonicated three ×30 s (Fisher Scientific D100 Sonic Dismembrator, output power 7 W) and centrifuged at 27,000*g* for 30 min at 4 °C. The resulting supernatant was incubated with 30 ul of (RIPA-equilibrated) streptavidin-sepharose beads (GE) with end-over-end rotation for 2 h at 4 °C. Beads were washed 7×1 mL 50 mM ammonium bicarbonate (pH 8.0) prior to tryptic digest.

### 1.2 BioID in cultured cells

The FlagBirA⁎ or FlagBirA⁎-MYC 293 cells (at ~70% confluence) were treated with 1 μg/ml tetracycline for 24 h. Cells were scraped into PBS, pooled, washed twice in 25 ml PBS, and collected by centrifugation at 1000*g* for 5 min at 4 °C. Cell pellets were lysed in 5 mL ice-cold modified RIPA buffer. 250 U benzonase (EMD) was added, and biotinylated proteins isolated as above.

### 1.3 Mass spectrometry

One microgram of MS-grade TPCK trypsin (Promega, Madison, WI) dissolved in 70 μl of 50 mM ammbic (pH 8.3) was added to the streptavidin-sepharose beads and incubated at 37 °C overnight. The eluate was collected and beads washed twice in 100 ul 50 mM ammonium bicarbonate. The combined eluate was lyophilized and brought up in 0.1% formic acid. Liquid chromatography analytical columns (75 um inner diameter) and pre-columns (150 um inner diameter) were made in-house from fused silica capillary tubing from InnovaQuartz (Phoenix, AZ) and packed with 100 Å C18-coated silica particles (Magic, Michrom Bioresources, Auburn, CA). Peptides were subjected to nanoflow liquid chromatography – electrospray ionization – tandem mass spectrometry (nLC-ESI-MS/MS), using a 95 min reversed phase (10–40% acetonitrile, 0.1% formic acid) buffer gradient running at 250 nL/min on a Proxeon EASY-nLC pump in-line with a hybrid linear quadrupole ion trap (Velos LTQ) Orbitrap mass spectrometer (Thermo Fisher Scientific, Waltham, MA). A parent ion scan was performed in the Orbitrap, using a resolving power of 60,000. Simultaneously, up to the twenty most intense peaks were selected for MS/MS (minimum ion count of 1000 for activation) using standard CID fragmentation. Fragment ions were detected in the LTQ. Dynamic exclusion was activated such that MS/MS of the same *m*/*z* (within a 10 ppm window, exclusion list size 500) detected two times within 15 s were excluded from analysis for 30 s.

For protein identification, Thermo. RAW files were converted to the .mzXML format using Proteowizard [2], then searched against Human RefSeq Version 45 (appended with cRAP and reversed decoy database based on Refseq v45) using the MASCOT [3] and Comet [4]. Search parameters specified a parent MS tolerance of 15 ppm and an MS/MS fragment ion tolerance of 0.4 Da, with up to two missed cleavages allowed for trypsin. Oxidation of methionine and ubiquitylation of lysine residues were allowed as variable modifications. Each AP was analyzed using at least two technical replicates. Statistical validation of peptide and protein identifications was performed using iProphet [5] as part of the trans-proteomic pipeline [6,7]. For each search, the iProphet probability at 1% error rate was used as a cutoff value to generate SAINT-compatible input files [8,9]. SAINT parameters were as follows: 5000 iterations, low mode off (0), minFold 1 and normalization On (1) [9]. Interactors with a 90% confidence level are reported, and the average peptide counts per two technical runs shown.
